# “Clinicopathological features and treatment outcomes of differentiated thyroid cancer in Saudi children and adults”

**DOI:** 10.1186/s40463-015-0102-6

**Published:** 2015-11-06

**Authors:** Khalid Hussain AL-Qahtani, Mutahir A. Tunio, Mushabbab Al Asiri, Naji J. Aljohani, Yasser Bayoumi, Khalid Riaz, Wafa AlShakweer

**Affiliations:** Department of Otolaryngology-Head & Neck Surgery, College of Medicine, King Saud University, Riyadh, Saudi Arabia; Radiation Oncology, King Fahad Medical City, Riyadh, Saudi Arabia; Radiation Oncology, Comprehensive Cancer Center, King Fahad Medical City, Riyadh, 59046 Saudi Arabia; Endocrinology and Thyroid Oncology, King Fahad Medical City, Riyadh, 59046 Saudi Arabia; Radiation Oncology, NCI, Cairo University, Cairo, Egypt; Histopathology, Comprehensive Cancer Center, King Fahad Medical City, Riyadh, 59046 Saudi Arabia

**Keywords:** Differentiated thyroid cancers, Children, Adults, Clinicopathological characteristics, Treatment outcomes

## Abstract

**Introduction:**

Age is an important prognostic factor in differentiated thyroid cancer (DTC). Our aim was to evaluate differences in clinicopathological features and treatment outcomes among children and adult patients with DTC.

**Materials and methods:**

We studied 27 children (below 18 years) with DTC treated during the period 2000–2012 and were compared with (a) 78 adults aged 19–25 years and (b) 52 adults aged 26–30 years treated during the same period in terms of their clinicopathological features and long term treatment outcomes. Locoregional recurrence (LRR), locoregional control (LRC), distant metastasis (DM), distant metastasis control (DMC), disease free survival (DFS) and overall survival (OS) rates were evaluated.

**Results:**

Mean age of children was 13.5 years (range: 5–18), while mean age of adults was 24.6 years (range: 19–30). In children, female: male ratio was 2.85:1, and in adults female: male ratio was 7.1:1 (*P = 0.041*). No significant difference in tumor size was seen between the two groups (*P = 0.653*). According to American Thyroid Association (ATA) risk stratification classification, the children (85.2 %) were found to have at high risk as compared to adults *P = 0.001*. Post-thyroidectomy complications and RAI induced toxicities were observed more in children than adults (*P = 0.043* and *P = 0.041* respectively). LRR occurred in 6 (22.2 %), 9 (11.5 %) and 3 (5.8 %) in age groups of <18 years, 19–25 years and 26–30 years respectively (*P = 0.032)*; while DM was seen in 10 (37.0 %), 9 (10.3 %) and 5 (9.6 %) in age groups of <18 years, 19–25 years and 26–30 years respectively (*P = 0.002)*. Ten year DFS rates were 67.3 % in age group below 18 years, 82.4 % in age group of 19–25 years and 90.1 % in age group of 26–30 years (*P = 0.021)*.

**Conclusion:**

At the time of diagnosis, children with DTC were found to have more aggressive clinicopathological characteristics. Comparatively lower LRC, DMC and DFS rates in children warrants further multi-institutional studies.

## Background

The incidence of differentiated thyroid cancers (DTC), including papillary (PTC) and follicular (FTC) variants, is increasing exponentially over the past years throughout the world with a wide geographic variation [1]. In Kingdom of Saudi Arabia, DTC is the second most common malignancy among middle aged women [2]. DTC is relatively uncommon in children (age 18 years or below), adolescents (under 25 years old), and young adults (above 25 and below 30 years) accounting for 3–10 %. Recent data has suggested that the frequency of DTC in children varies according to pre-puberty, puberty and adolescence growth phases [3, 4]. In contrast to adults, DTC in pre-pubertal children has some distinctive differences; such as (a) larger primary tumor at the time of diagnosis; (b) high prevalence of neck lymph nodes and distant metastases at the time of diagnosis; and (c) the high risk of recurrences [5].

DTC in children and adolescents is treated in similar fashion as that in adults, primarily because of rarity of disease in pediatric population and lack of availability of pediatric DTC treatment guidelines [6]. Fortunately, even with aggressive behavior, DTC in children has excellent prospects with use of thyroidectomy, neck dissection, radioactive iodine-131 (RAI) ablation and suppression of thyroid-stimulating hormone (TSH) secretion with levothyroxine [5, 6].

In the current study, we designed our approach towards the comparative analysis of different clinicopathological features and treatment outcomes among children, adolescents and young adults with DTC in Kingdom of Saudi Arabia.

## Methods

After formal approval from the institutional ethical committee, medical records of 157 DTC patients of age less than 30 years, who were treated at our hospital during the period of July 2000 and December 2012 were reviewed using computer based database system.

For the purpose of present study, the study population was divided in three groups; (a) children (patients below or equal to the age of 18 years), (b) young adults (aged 19 to 25 years) and (c) adults (aged 26 to < 30 years) [7].

### Demographic, clinicopathological and radiological data

Demographic and clinical data including age at the time of diagnosis, gender and symptomatology were reviewed. A detailed second review of all histopathological specimens was done by experienced histopathologist. Different histopathological characteristics, including tumor size, histopathologic variants, multifocality, tumor, lymph node and metastasis (TNM) staging were recorded. Data was collected from different imaging modalities including neck ultrasonography (USG), whole body I-131 scintigraphy (WBS), computed tomography (CT) scan of neck and chest and flourodeoxyglucose positron emission tomography (FDG-PET). Data regarding different treatment modalities including thyroidectomy, +/− neck dissection, adjuvant radioactive iodine-131 (RAI) ablation and its doses in millicurie (mCi) were also recorded.

### Statistical analysis

The primary endpoint was the disease free survival (DFS). Secondary points were; the comparative analysis of different clinicopathological features of DTC in children and adults, locoregional control (LRC), distant metastasis control (DMC) and overall survival (OS) rates.

Local recurrence (LR) was defined as the duration between surgery date and date of clinically or radiologically detectable disease in the thyroid bed and/or in cervical lymph nodes on imaging (USG, WBS, CT and FDG-PET) after evaluation of elevated thyroglobulin (TG) levels. Distant metastasis (DM) was defined as the duration between surgery date and date of documented disease outside the neck on imaging after evaluating for elevated TG. DFS was defined as the duration between surgery date and date of documented disease reappearance/relapse, death from cancer and/or last follow-up. OS was defined as the duration between surgery date and date of patient death or last follow-up.

Chi-square or Student’s t-tests were used to determine the differences in various clinical variables. Probabilities of LRC, DMC, DFS and OS rates were shown with the Kaplan-Meier method and the comparison for various survival curves was performed using log rank. All statistical analyses were performed using the computer program SPSS version 16.0.

## Results

### Demographic and clinicopathological features of cohort

The mean age at the time diagnosis of whole cohort was 22.7 years (range: 5–30). The whole cohort (*n* = 157) was consisted of 134 (85.4 %) females and 23 (14.6 %) males. Male gender was predominant in children (25.9 %) than that in groups 19–25 years (10.3 %) and 26–30 years (15.4 %) respectively *P = 0.038*. The predominant histopathology was PTC (95.5 %) whereas FTC was seen only in 4.5 % of patients. Mean tumor size was 2.9 cm (range: 0.4–6.5) without any statistically significant difference between the children and adults (*P = 0.653*). In contrast to adults, positive lymph nodes were more evident in the children (74.1 %), *P = 0.012*. According to ATA risk stratification classification, the children (85.2 %) were found to be at higher risk as compared to other age groups *P = 0.003*. The clinicopathological and treatment characteristics are described in Table 1.

**Table 1:** Comaprison of clinicopathological and treatment characteristics of children and adult patients with differentiated thyroid carcinoma at presentation

Variables	Children with DTC (<18 years)	Young adults with DTC (19–25 years)	Adults with DTC (26–30 years)	*P* value
	(*n* =27)	(*n* =78)	(*n* =52)	
Age (years)				
Mean/range/SD	13.5 (5–18) ± 4.3	22.41 ± 3.3	27.5 ± 2.8	0.0001
<10 years	8 (29.6)	0	0	
11–18 years	19 (70.4)	0	0	
Gender, n (%)	ᅟ	ᅟ	ᅟ	ᅟ
M	7 (25.9)	8 (10.3)	8 (15.4)	0.038
F	20 (74.1)	70 (89.7)	44 (84.6)	0.160
Tumor size (cm)	ᅟ	ᅟ	ᅟ	ᅟ
Mean/range/SD	2.8 (0.8–6.5) ± 1.36	2.8 (0.8–6.4) ±1.36	2.9 (0.4–6.5) ±1.62	0.653
Histology n (%)	ᅟ	ᅟ	ᅟ	ᅟ
Papillary	27 (100)	72 (92.3)	51 (98.1)	0.605
Follicular	0	6 (7.7)	1 (1.9)	
T stage, n (%)	ᅟ	ᅟ	ᅟ	ᅟ
T1	12 (44.4)	27 (34.6)	26 (50.0)	0.860
T2	11 (40.8)	34 (43.6)	15 (28.9)	
T3	4 (14.8)	15 (19.2)	10 (19.2)	
T4	0	2 (2.6)	1 (1.9)	
N stage, n (%)	ᅟ	ᅟ	ᅟ	ᅟ
N0	7 (25.9)	42 (53.8)	32 (61.5)	0.012
N1	20 (74.1)	36 (46.2)	20 (38.5)	
N1a	9 (45.0)	22 (61.1)	11 (55.0)	
N1b	11 (55.0)	14 (38.9)	9 (45.0)	
M stage, n (%)	ᅟ	ᅟ	ᅟ	ᅟ
M0	26 (96.3)	77 (98.7)	52 (100)	0.379
M1	1 (3.7)	1 (1.3)	0	
LVSI, n (%)	ᅟ	ᅟ	ᅟ	ᅟ
Yes	12 (44.4)	27 (34.6)	16 (30.7)	0.031
No	15 (55.6)	51 (65.4)	36 (69.3)	
Multifocality, n (%)	ᅟ	ᅟ	ᅟ	ᅟ
Yes	9 (33.3)	43 (55.2)	32 (61.5)	0.012
No	18 (66.6)	35 (44.8)	20 (38.5)	
Risk stratification, n (%)	ᅟ	ᅟ	ᅟ	ᅟ
Low	2 (7.4)	17 (21.8)	11 (21.1)	0.003
Intermediate	2 (7.4)	23 (29.5)	21 (40.4)	
High	23 (85.2)	38 (48.7)	20 (38.5)	
Surgery, n (%)	ᅟ	ᅟ	ᅟ	ᅟ
Total thyroidectomy	23 (85.2)	68 (87.2)	44 (84.6)	0.065
Near total thyroidectomy	4 (14.8)	10 (12.8)	8 (15.4)	
Neck dissection, n (%)	ᅟ	ᅟ	ᅟ	ᅟ
No	3 (11.1)	25 (32.1)	15 (28.8)	0.047
Yes	24 (88.9)	53 (67.9)	37 (71.2)	
Central	13 (54.2)	30 (56.6)	18 (48.6)	
Lateral	11 (45.8)	23 (43.4)	19 (51.4)	
Adjuvant RAI therapy, n (%)	ᅟ	ᅟ	ᅟ	ᅟ
No	1 (3.7)	7 (9.0)	2 (3.8)	0.460
Yes	26 (96.3)	71 (91.0)	50 (96.2)	
30 mCi	0	4 (5.6)	1 (2.0)	
100 mCi	7 (27.0)	20 (28.2)	19 (38.0)	
150 mCi	14 (53.8)	36 (50.7)	20 (40.0)	
200 mCi	5 (19.2)	11 (15.5)	10 (20.0)	

*N* number, *DTC* differentiated thyroid cancers, *SD* standrad deviation, *M* male, *F* female, *T* tumor, *N* node, *M* metastasis, *LVSI* lymphovascular space invasion, *RAI* radioactive iodine, *mCi* millicurie

All patients underwent total or near total thyroidectomy without any difference between children and adults. However, because of palpable or radiological visible lymph nodes, the neck dissection was attempted more in children than those in other age groups (88.9 % vs. 67.9 % vs. 71.2 %) *P = 0.047*. Among 157 patients, 147 (93.6 %) patients were given adjuvant RAI ablation. Median time to RAI ablation was 8.2 weeks (6.8–16.6) from thyroidectomy and no significant differences in the frequency of adjuvant RAI ablation were observed between the children and adults (*P =0.460*).

### Complications and Toxicities

Post-thyroidectomy complication rates were minimal; however, permanent hypocalcemia was statistically and significantly high in children and young adults (*P = 0.043*). Overall, RAI ablation was tolerated well without any grade 3 or 4 side effects; however, acute and late (any grade) complications were seen significantly higher in children and young adults (*P = 0.041*) Table 2.

**Table 2:** Comparative analysis of complications of surgery and toxicities of radioactive iodine ablation in our cohort

Toxicity	Children Age below 18 years	Young adults Age 19–25 years	Adults Age26-30 years	*P* value
	(*n* = 27)	(*n* =78)	(*n* =52)	
Thyroidectomy, n (%)				
Hypocalcemia	5 (18.5)	11 (14.1)	8 (15.4)	0.064
Transient	4 (80.0)	9 (81.8)	7 (87.5)	
Permanent	1 (20.0)	2 (18.2)	1 (12.5)	0.043
Recurrent laryngeal nerve damage	1 (3.7)	1 (1.3)	1 (1.9)	0.072
RAI ablation, n (%)				
Acute:	16 (59.3)	36 (46.1)	18 (34.6)	0.041
Sialadenitis	5 (31.3)	12 (33.3)	6 (33.3)	0.071
Acute sickness (nausea, vomiting)	4 (25.0)	17 (47.2)	10 (55.5)	0.042
Neck pain	7 (43.7)	7 (19.5)	2 (11.1)	0.001
RAI ablation, n (%)				
Late:	2 (7.4)	3 (3.8)	1 (1.9)	0.034
Sicca syndrome	1 (50.0)	3 (100)	0	
Nasolacrimal duct obstruction	1 (50.0)	0	1 (100)	
Second malignancy	0	0	0	
Infertility	0	0	0	
Lung fibrosis	0	0	0	

*RAI* Radioactive iodine, *n* number

### Treatment outcomes

Median follow-up period was 6.2 years (range: 2.0–10). For whole cohort, the 10-year LRC and DMC rates were 87.2 and 84.2 % respectively. The 10-year LRC and DMC rates were significantly lower in children (75.3 and 64.7 % respectively) than those young adults and adults. Total 18 LRs (11.5 %) were observed; 6/27 in children, 9/78 in young adults and 3/52 in adults (*P = 0.032*) Table 3. The LRs were salvaged by surgery; lateral neck dissection (13 patients); completion thyroidectomy (4 patients) and excision (2 patients) followed by RAI ablation (16 patients). Similarly, total 24 DM (15.3 %) were observed; 10/27 in children, 9/78 in young adults and 5/52 in adults (*P = 0.002*). DMs were salvaged by RAI ablation and palliative irradiation for bony lesion (one patient).

**Table 3:** Pattern of failure after thyroidectomy and radioactive iodine therapy in children and adults with differentiated thyroid cancer

Failures	Children Age below 18 years (*n* = 27)	Young adults Age 19–25 years (*n* = 78)	Adults Age 26–30 years (n =52)	*P* value
Locoregional, n (%)	6 (22.2)	9 (11.5)	3 (5.8)	0.032
Thyroid bed	1 (16.7)	2 (22.2)	0	
Lymph nodes	5 (83.3)	7 (77.8)	3 (100)	
Distant, n (%)	10 (37.0)	9 (10.3)	5 (9.6)	0.002
Lungs	10 (100)	9 (100)	4 (80.0)	
Bones	-	-	1 (20.0)	
Locoregional + distant	4 (14.8)	4 (5.1)	2 (3.8)	0.035
10 year LRC rate	75.3%	87.3 %	93.2 %	0.001
10 year DMC rate	64.7 %	84.8 %	94.2 %	0.0001
10 year OS rate	92.3 %	96.2 %	100 %	0.079

*n* number, *LRC* locoregional control, *DMC* distant metastasis control, *OS* overall survival

**Figure. 1:**
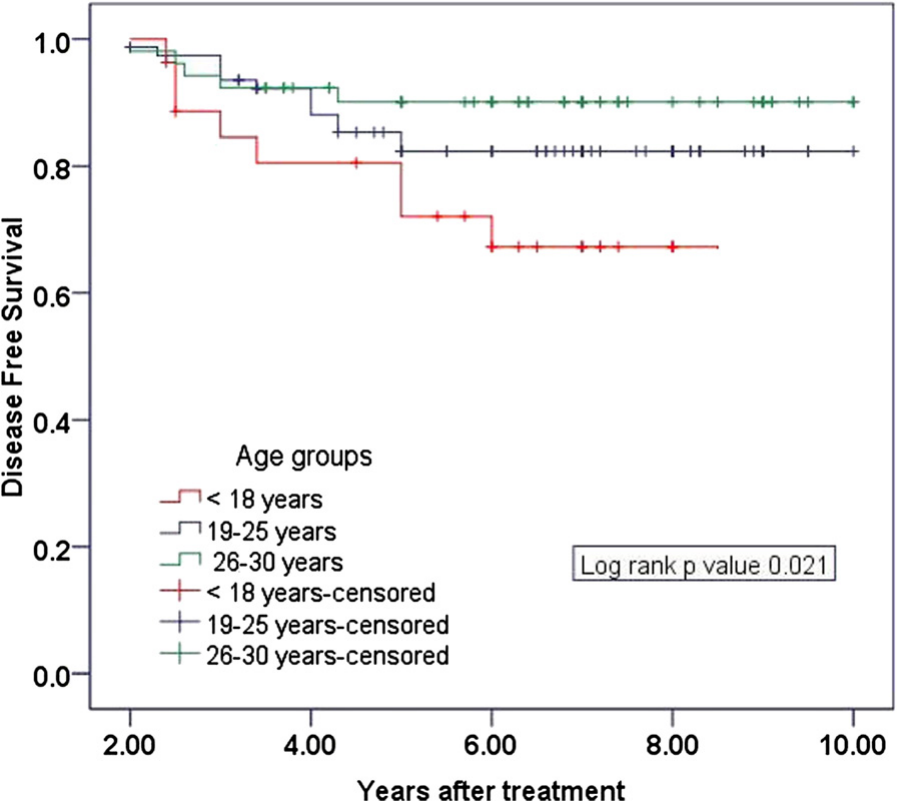
Kaplan-Meier curves of disease free survival in age groups < 18 years, 19–25 years and 26–30 years

The 10-year DFS rates were 67.3 % in children, 82.4 % in young adults and 90.1 % in adults (*p = 0.021*) Fig. 1. There was no statistically significant difference in 10-year OS rates among all age groups (*P = 0.075*).

## Discussion

Due to rarity of DTC in children and young adults, few major controversies regarding aggressiveness of clinical behavior and optimal treatment still persist [8]. In present study, some unusual observations related to histopathological features were observed, which are in contradiction to reported literature.

Firstly, no statistically significant difference in primary tumor volumes or presence of extrathyroidal extension (ETE) was observed between the children and adults. Previous studies have hypothesized that the tumor volumes tend to be relatively larger in children when compared to adults, probably attributed to smaller thyroid volume in children, thus higher chances of ETE and capsular invasion [3–5, 9, 10]. Similar to our results, few studies have also reported no correlation between tumor volumes and pediatric population [11–13]. However, no difference in tumor volumes or ETE among children and adults can be criticized for relatively few pre-pubertal children in our cohort. Secondly, in contrast to children, multifocality was found more prevalent in our adult cohort, which is a reasonable argument against wide practice of total thyroidectomy as primary surgical approach in children [14, 15] Thirdly, male gender was predominant in our pediatric cohort, which is likely attributed to hormonal changes during pre-puberty and puberty [16].

Further, it was also clear that larger percentage of children underwent elective neck dissections mainly because of increased frequency of clinical or radiological cervical lymphadenopathy at the time of diagnosis. Similarly, treatment related complications were observed more in children. Large percentage of permanent hypocalcemia supports the notion of less aggressive surgery (lobectomy or hemi-thyroidectomy) for primary tumor in children, to decrease the risk of surgical complications [17, 18]. Similarly, the children experienced relatively more acute and late (any grade) RAI induced complications in our cohort. However, no RAI ablation induced second primary malignancy (SPM), lung fibrosis or infertility was observed. Possible explanation could be the short follow-up, and given lower cumulative doses of RAI in our cohort [5, 19].

In contrast to previously published data, children in our cohort experienced relatively higher all-sites recurrence rates, thus lower 10-year DFS rate (67.3 %) after extensive treatment [4, 5, 7, 9, 20, 21] which is attributed to our ATA high risk (85.2 %) pediatric cohort.

There were few limitations of our study. Firstly, since more children were rendered to neck dissections, there was a possible selection bias when it came to the number of lymph nodes found to harbor metastasis. Secondly, TSH suppression therapy complications including effects on child growth, osteoporosis and cardiovascular diseases were not studied, as children are still in growing phase, and TSH suppression therapy theoretically may affect their final height [22]. Thirdly, we did not look into molecular patterns in childhood DTC. Bongarzone I et al. initially reported that the frequency of ‘Rearranged during Transcription’ (RET) and ‘neurotrophic tyrosine kinase receptor-1’ (NTRK1) oncogenic activation is significantly higher in childhood DTC, thus contributing to childhood DTC carcinogenesis [23]. Besides RET/NTRK1, over-expression of proto-oncogenes ‘MET’, and ‘vascular endothelial growth factor’ (VEGF) have been found to cause high recurrence rate in children [24].

## Conclusions

In conclusion, DTC in Saudi children is far more aggressive entity in nature as compared to adults. Children are at higher risk of treatment (thyroidectomy and RAI) related complications as compared to adults. Lower LRC, DMC and DFS rates in children after extensive therapy of thyroidectomy, neck dissection followed by RAI, TSH suppression warrants further multi-institutional studies.
